# The Amalgam Question and Dentists with “Yankee Wit”

**Published:** 1898-06

**Authors:** Charles H. Taft

**Affiliations:** Boston, Mass.


### THE AMALGAM QUESTION AND DENTISTS WITH "YANKEE WIT."[1]

BY CHARLES H. TAFT, A.B., D.M.D., BOSTON, MASS.

Mr. President and Fellows of the Academy,—One of the incidents of a recent dental meeting in this city calls to mind an excellent paper entitled "Character as demanded in Professional Life," by our esteemed friend, Dr. Eddy, published in the July, 1893, issue of the International Dental Journal. And the thought occurs that there are times in our professional careers when a re-reading of such papers might be of use if we are willing to profit by the helpful suggestions with which this particular paper abounds, and by recognizing the truth of what Dr. Eddy calls the

[1] Read before the American Academy of Dental Science, Boston, January 5, 1898.

"morally painful axiom 'that character in every department of life and action is a good investment,'—that, in fact, 'it pays.'"

It would be difficult for me to emphasize more emphatically this axiom than Dr. Eddy has done, whether it concerns us as dentists or men in other callings or walks of life. But the fact that there are times when men in dental meeting, as well as in private practice, appear to wink at, if not to openly countenance, questionable methods of practice, implying an utter disregard for the existence of any moral law, may well cause us to reflect how important it is to avoid, as Dr. Eddy has said, "by the slightest word or act anything which shall call down reproach upon our profession or upon us as men." The incident to which I have reference was in connection with the discussion of the subject entitled "A Consideration of the Objections offered by Physicians to Amalgam Fillings."

In the effort to show that the statements made by many physicians relative to the at times harmful effects of amalgam were not worthy of serious attention, one of the essayists advanced the argument that these fillings never had been and never could be a source of harm to any one, for the reason that dentists have been filling teeth with this material for sixty years with no serious results, so far as he knew. Any belief on the part of the patient that amalgam fillings were doing him harm the essayist felt was due entirely to the imagination, as could be abundantly proved by the use of a little strategy; and, as an illustration of what I regret to believe is a common practice with some in our profession who are unwilling to investigate in any dignified or thoughtful manner the objections offered to amalgam, the essayist cited a case where "a dentist, making use of a little Yankee wit, had said to a lady who wished to have an amalgam filling removed because it made her sick, 'I will replace this amalgam with silver,' and did so, the result being that she had no further trouble with it."

With the feeling that a subterfuge like this detracted from rather than added to the force of the arguments which the essayist advanced, its recital called to mind two somewhat similar cases in my own practice the past year, the first being that of a lady who said to me as I made an examination of her mouth, "My physician wishes me to tell you not to put any amalgam into my teeth." "I presume you know that you already have several such fillings," I said, upon making the examination. "Impossible!" she exclaimed. "Why, it is only three months since I went to Dr. ——, who put all those fillings in. I told him what I have just said to you concerning my own and the wishes of my physician, and kept saying to him, 'Now, doctor, you are not filling my teeth with anything that has mercury in it, are you?' 'Oh, no,' the dentist replied, 'there is nothing in this material that will prove of the slightest harm or injury to you.' "

To make a long story short, the lady was put to the pain, time, and further expense of having the fillings removed, simply because one of these "black sheep" in our profession, a *dishonest man*, with or without Yankee wit, caring nothing for his own honor and reputation or for his patient's welfare, was, presumably, too lazy and too ignorant to set about ascertaining in any intelligent manner whether the objections offered by the physician in this case were well founded or not.

Now, I submit that any man who, thinking to fool both patient and physician, will deliberately charge and accept a fee for a dishonest service like that fails to appreciate the obligations of his profession, besides placing himself in a very unenviable position.

The second was that of a gentleman who came to me as a new patient. Noticing, upon examination of his mouth, what an exceptionally fine set of teeth he had, and the presence of three or four amalgams where gold could as easily have been inserted, I asked through curiosity why his former dentist had elected to fill such simple cavities with amalgam. He replied, with much surprise, that he was entirely unaware of the presence of any amalgam in his teeth; that he had expressly forbidden his dentist to use it, and the latter had, as it proved, ignored his wishes. The patient left my office with the declaration that "he would make it warm for that man," and with an emphasis that left no doubt of his purpose to make good his word.

Now, gentlemen, my only excuse for bringing this subject before you is for the reason that I have come to take a great interest in it myself, and because I believe this Academy is both able and willing to discuss it intelligently. And because, furthermore, of an ever-growing conviction on my part that we have not begun to appreciate how many people at one time or another become susceptible to mercury, or whose systems are already poisoned from the effects of mercurial medicines in one form or another. Few of us as dentists, I believe, are able to recognize cases of either mercurial susceptibility or poisoning even though there may be abundant evidence standing out, as we say, "as plain as the nose on one's face," and instantly recognized by all who are familiar with symptoms of mercury-poisoning.

I do not hesitate to admit that I once had the same honest conviction I know many of you still have that a large class of physicians are entirely in error in the position they have taken relative to amalgam fillings. For a long time I found amalgam too convenient and useful a material in the saving of teeth to practically discard it because of what seemed to me mere whims on the part of physician or patient, and I did not feel at all troubled in a continued use of it, though I knew there was an ever-increasing class of physicians and laymen all over the country who objected to it.

I shall not attempt to convince any one by the use of Yankee wit or sharp practices that the harmful properties of mercury do not necessarily become inert or eliminated when the metal is combined with one or more metals in the form of amalgam, but will ask you to follow me in my method of considering the question, and if there is anything of sophistry in such reasoning as I have to advance I trust no one will hesitate to make it plainly apparent.

Let me begin by repeating a statement which some one has made, and to which I think we can all assent, that "what is one man's meat is another's poison;" for within this statement lies, it seems to me, the key to a solution or to a better understanding of the question. In other words, we are forced to admit that there is a susceptibility in all persons to different drugs or noxious influences varying both in degrees and kinds.

Now, what do we mean by *susceptibility?* Let me offer a definition in the words of one of our friends in the medical profession that "it is an abnormal state of the system, owing to the presence of such morbid forces (generally inherited) which have the quality of attracting other and similar forces, and whose activity is in direct ratio to the lessened systemic resistance." This gentleman goes on to say that "drug forces in the organism follow the same law as acute morbid forces (when uncomplicated) in being self-limited. When drug forces produce an effect upon diseased conditions it is because there exists a certain degree of similarity between them. Drugs aggravate, ameliorate, or change diseased conditions when they have reference to quantity, degree of similarity, and plane of action, while the high dynamizations, instead of antidoting the effects of crude drugs, more nearly comply with the conditions of the sick-producing causes. Man is never made sick any length of time by a drug force unless he presents a susceptibility to its action, and this only proves a similarity of the drug to his condition, which may be curative in the proper potency." Let us consider these statements somewhat collectively and see if they are true.

Take a simple illustration. To a majority of people the common strawberry is a delicious article of food. To a minority it is a violent poison. How do we know this? Not because some one says so, but because we have seen cases where an indulgence in the fruit brings on at once well-marked and characteristic symptoms of distress. By taking note of and systematically recording every abnormal symptom in any part of the system of one who is susceptible to the poisonous properties of the berry we shall acquire definite knowledge as to the physiological action. We shall find that in one person it produces but a single symptom,—a spasm or contraction in the muscles of the throat, which prevents a swallowing of the berry,—while in another person it produces a violent itching and burning sensation in different parts of the body, acute pains and more or less functional disturbance in general. It may affect no two persons alike, and yet when we compare the abnormal symptoms in their totality which the berry produces in a number of people who are susceptible to its toxic properties we find it has very marked characteristics that give it an individuality among drugs when considered as poisons.

Now, drugs which have toxic properties generally have medicinal properties. This is true of the strawberry, and the success which the physician of either of the homœopathic or allopathic school of medicine has in ameliorating or changing diseased conditions lies, I believe, in his ability to meet these conditions upon *similar planes of action.* Herein, too, lies the difficulty with physicians of either school in selecting that drug which, in its medicinal form and upon a similar plane of action, will successfully overcome and cure such conditions.

Now, while we are using the strawberry in illustration, let me say that I have among my patients a lady who from childhood was unable to eat strawberries in any form, but who could, after being *cured* with the same agent in its medicinal form, eat them with impunity. Do not infer from this by any means that the berry in its medicinal form is anything like an invariable specific for the cure of those who may be susceptible to its poisonous properties. That is an error into which many of us fall when we say that if, according to the law of similars, "like cures like," then a dose of strawberry in the medicinal preparation ought to cure one who is poisoned by it. Having, perhaps, tried it, and finding it does *not* always cure, we say the law is a humbug, and those who practise medicine in accordance with such a so-called law of cure are equal if not greater humbugs.

In the case just mentioned there happened to be a perfect likeness or similarity between the diseased condition and the medicinal action of the drug. If, as in other cases, there had been found to exist but a *partial* similarity, its effect in such instance would have proved only *palliative* or *partially* curative, and it would have been necessary to employ some other drug which would have entirely changed or removed the abnormal symptoms. That drug alone which is found to meet the totality of the symptoms will have the desired curative action. It is not a question for us to consider whether the drug be given in the crude or dynamized form to get the best results.

If we can keep in mind that it is always a disturbed condition of the *vital force*, never the material organs and tissues of the body themselves, upon which the action of medicines is to be directed (the restoring of its equilibrium, in other words) we can understand, if we give the matter any thought whatever, that this vital force must be in its very essence a spirit-like force, and that it must be met with a similar force upon a similar plane, when it becomes necessary to restore its equilibrium.

I have used the strawberry as a convenient illustration of taking any drug we please and studying its properties and action in the same manner. In no different way may mercury be studied. I have heard it said in dental meeting that crude mercury is inert, and that in this form a man could take a pound or two without harm, incredible as this may seem. As a matter of precaution, however, I should urge the taking of crude mercury in a less heroic dose by those who desire to make special study of its physiological action. Personally, I should prefer to let the man who has a metallic lining to his stomach experiment with mercury in either one- or two-pound doses.

Substituting mercury, then, for the strawberry, let us consider for a moment some of its effects upon those who show a susceptibility to it.

I have among my patients a gentleman who cannot be in a room fifteen minutes where there is an uncorked bottle of the bichloride of mercury without experiencing a violent headache. In due season, after removal of the exciting cause, the headache disappears. This, to my mind, is conclusive evidence that the gentleman is susceptible to mercury. I should not have to remove his amalgams, if he had any, to be further convinced of the fact, and I assume that you would not ask me to demonstrate with scalpel and microscope the actual presence of mercury globules in the tissues and cells of his brain, and to furnish you in this way with some really scientific (?) proof that this gentleman is susceptible to and poisoned to a limited extent by mercury. I could not do it were I to try, nor could I prove it in the same manner if I stated that a patient who had chronic headaches was undoubtedly suffering from mercurial poisoning when a removal of the amalgams caused those headaches sooner or later entirely to cease. I have both the written and unwritten testimony of so many patients whose headaches have become a thing of the past with the removal of their amalgams that I am convinced these fillings very often prove a source of evil, —solely because of the mercury, understand me,—though I may not convince others with what appears to be conclusive evidence.

When headaches have reached the chronic stage I do not believe a radical cure is ever effected without good and sufficient cause. I believe the vital force is very often insufficient to combat the influence of a powerful drug force and to be affected by the right medicinal force until the obstruction to its curative action has been removed. In the case of the gentleman to whom I have referred, I have shown that the drug force was sufficiently powerful to throw the vital force out of equilibrium, and I feel confident that had he any amalgam fillings in his teeth a headache would not clear up so quickly as it now does with the removal of the bottle of bichloride.

We know very well that when a dead pulp causes an abscess to form, with a constant discharge of pus from a fistulous opening, no permanent cure of abscess or fistula is possible until the exciting cause acting as an obstruction is removed. We can with well-chosen remedies which control the suppurative process often palliate or cause a temporary cessation of the discharge just as it is possible to relieve headaches in the same manner. But until the exciting cause has been entirely removed a complete cure cannot be said to have been effected.

We may argue, if we please, that cessation or cure of chronic headaches upon removal of amalgam fillings does not prove these fillings or the mercury to be the exciting cause, or that they often act as an obstruction to the action of medicines. No more, then, does the fact that a fistula heals upon the removal of a dead pulp and subsequent treatment prove either the dead pulp to be the exciting cause or that *it* may often act as an obstruction to the action of drugs which have power to control the suppurative process. No more does a cessation of toothache by the application of aconite and iodine, oil of cloves, or other drugs prove that in some mysterious manner such remedies have had a palliative, if not a curative action, and been the means of restoring to the vital force its equilibrium. Much of our knowledge comes necessarily from both inductive and deductive reasoning, and is none the less scientific when by means of such reasoning we are able to systematically organize or classify it.

A very good evidence of a patient's susceptibility to mercury is the existence of a metallic taste in the mouth. I frequently hear patients complain of this without giving the matter a thought. How many of us do, in fact? Other evidences of mercurial poisoning are loss of appetite, followed by disturbance of the circulatory system, nervous irritability, and characteristic fetor of the breath. The gums about the necks of the teeth become inflamed, spongy, bleeding, ulcerated, and sore to touch. Salivary glands inflamed, swollen, and also sensitive to touch. Secretion of saliva is augmented and it becomes of a ropy consistence, with offensive taste and smell. An interesting symptom is that of the tongue becoming coated with a white slime and bearing upon its edges the imprint of the teeth. I shall refer to this presently.

Mercury has an affinity for all mucous membranes, especially of the mouth, throat, and nose, producing in the latter great congestion and catarrhal inflammation. It is in cases of chronic catarrh particularly that physicians who are careful and accurate prescribers find themselves so often hampered and baffled in their efforts to cure the patient until all amalgam fillings have been removed. Even then it often requires time to effect a permanent cure owing to the difficulty in removing entirely the poisonous effects of the metal from such systems as have become more thoroughly permeated with it.

The manifestations of mercurial poisoning upon the mucous membranes of the mouth, throat, and tonsils are too well known to be mistaken by one who is familiar with them; though without giving the matter the slightest thought or study we are quite apt to argue that the physician is confounding such inflammations or ulcerations with fistulæ of alveolar abscess or with inflammations of tissue caused by the ragged edges of amalgam fillings inserted by some careless or slovenly operator.

Oftentimes a susceptibility to mercury is the result of long exposure, while again it may be brought about in a very short time. Sometimes the influence of mercury falls upon a single part of the system,—the nervous, for instance,—when as vapor it finds its way to the blood through the lungs, resulting in a paralytic condition.

I remember reading of a case where two barometer-makers became badly poisoned by sleeping one night in a room where there was a pot of mercury on the stove. One was severely salivated, the other contracted a shaking palsy from which he never recovered.

Rather exceptional cases like these, however, do not prove a general susceptibility to mercury, nor that the majority of barometer- or mirror-makers are poisoned to any alarming extent from continued exposure to the vapor of mercury, any more than the fact that now and then some one working in a match-factory and developing phosphorus-necrosis of the maxillary bones establishes proof of a susceptibility to phosphorus in the majority of factory hands employed.

The symptom of the tongue bearing upon its edges the imprint of the teeth calls to mind a patient whose amalgams I removed while practising in Chicago. An extract from a recent letter in which the patient gives a report of himself may be of interest.

"While the amalgam fillings were in my teeth," he writes, "I had a decided and ever-present metallic taste in my mouth, and on arising in the morning my mouth seemed filled with this taste, which no amount of cleansing with brush and powder would wholly take away. This, however, disappeared with the amalgams. My tongue also showed the imprints of the teeth and does some yet, but they are going away slowly." This gentleman had been a sufferer from chronic headaches, bleeding piles, and other troubles, "all of which," he writes, "have disappeared for good." He adds, "I am now feeling fine in every way."

The fact that the system is often a good while in ridding itself from mercurial taint is well illustrated by this particular case. Sooner or later his tongue will show no imprints of the teeth whatever.

Now, this patient has not the *slightest* knowledge, so far as I know, that the symptom just mentioned is a strongly characteristic one of mercury. How many of us would recognize it as such? Not one dentist in fifty, I will venture to say, would recognize that or a great many other symptoms which a physician is quick to perceive as clearly characteristic of mercury. This, too, is but one out of perhaps many similar cases coming to all of us every day. An examination of the mouth revealing the presence of one or more amalgams affords us neither knowledge nor suspicion that the patient is susceptible to or, perhaps, already poisoned by mercury, whether to a greater or less degree it matters not. Judging by the kind of arguments I have heard advanced by some whose one object is, manifestly, to ridicule rather than to thoughtfully consider the objections offered to amalgam and to advance any dignified argument against these objections, I am certain it would not afford the *slightest* interest to such men even though there were unmistakable evidences of it. It is not always agreeable to recognize a *fact*, much less, perhaps, to admit it when recognized.

We say we never heard of any one who was poisoned or harmed by amalgam fillings. Perhaps there is good reason for this. Who does a patient consult when he is sick? Is it the dentist? It quite possibly might be if the patient considered the latter competent to either treat or cure him. He has confidence in our ability to put his teeth in good order and generally consults us for that purpose alone. But in our knowledge of diseased conditions and their obscure causes (with the exception of those pertaining to the oral cavity) the average layman, I maintain, has none, and *rightly* so. He would be justified in a similar lack of confidence in the physician's knowledge and skill in matters pertaining to our specialty. For that reason when in need of our services and advice he consults the dentist, not the physician.

But the physician who objects to amalgam on general principles does not deny that, considered as a filling-material alone, it serves a useful purpose in the saving of teeth. He does not assert that all people are at all times and under all conditions susceptible to mercury. He knows, however, that a susceptibility may be established at any time. He knows, as I, too, have learned, that when a drug force exerts a curative influence upon diseased conditions (or morbid forces) it is because there exists a *similarity between them.* If there is no effect, it must be for want of such similarity, as is the case when medicines are not well chosen, or because there is a stronger force acting either as an antidote or as an obstruction to the action of the remedy.

The objections offered to amalgam fillings by many physicians in both the leading schools of medicine seem to have stirred up a mingled interest, prejudice, and opposition among members of our profession. Many have shown and continue to manifest a desire to discuss the subject for the purpose of finding the truth. A few have argued and continue to assert, in spite of all evidence to the contrary, that the objections come *entirely* from physicians of the homœopathic faith, and for that reason alone are not worth considering, carrying their prejudices so far as to oppose the subject appearing for discussion on the programmes of several dental societies right here in the Commonwealth of Massachusetts, a State in which the right to freedom of thought and speech is, fortunately, seldom questioned. Such an opposition in *this* city or State by any member of the dental profession who courts such notoriety is too childish and silly to deserve one moment's serious attention.

From my view-point, the opinions of any man or set of men of recognized educational or professional attainment, who earnestly, conscientiously, and successfully devote their lives to the practice of medicine, are entitled to respect and thoughtful consideration. Personally, I respect and admire a physician of *any* school of medicine who, recognizing his inability to effect a cure in any particular case until all amalgam fillings have been removed, frankly tells the patient this. He shows himself at least to be too honest a man to continue taking his patient's money knowing he is not rendering a full equivalent for value received.

Do not misunderstand my position and assume that I would never under any condition sanction the use of amalgam. That it has its usefulness as a filling-material I freely admit, and for a long time I entertained the same thoughts and opinions concerning the objections to it that perhaps a majority of this Academy still do. With an independent study into the merits of these objections, the weighing of evidence as it has been presented to me by patients, and the willingness to recognize and admit as facts things which one could not dispute if he would, there has come a change of opinion on my part. But let no one infer for that reason that I wish to cast the slightest reflection in whatever I have said upon the opinions of any one who honestly differs with me. I do stand here, however, to encourage in *this* society—at least, never to discourage—an open, honest, manly discussion of this or any other subject that is of interest to us or to our patients. An ever-increasing number of men and women all over the country are discussing the question among themselves. Why not we? I believe, moreover, that when a patient tells us it is the wish of his physician that all amalgam fillings be removed, the work should be commenced and thoroughly and conscientiously carried through to the finish unless there is good reason for our wishing to first consult with the physician.

Far be it from me to pose as a moralist or to dwell at length upon the necessity of drawing fine distinctions between what a man's professional conscience tells him is right or wrong so far as his business dealings with patients is concerned. I only wish to emphasize my own disapproval of such methods of practice as those to which I have referred in the early part of this paper, and which, if generally employed, could not fail, and *justly* so, to bring discredit not only upon those who practised them, but upon a profession which we should wish to see honored, never disgraced. *An honest argument never needs the support of a dishonest trick.*

In conclusion, permit me to say that if I have learned anything which seems of value to me in the study of this question, it is my desire to give the benefit of it to others. The testimony which I have received from those who have experienced the benefits following removal of their amalgams furnishes me, at least, the pleasant satisfaction that in an unassuming way I am often doing what I can to *assist*, rather than *hamper*, the physician in that noblest of all works, the healing of the sick.

In whatever ways as individuals we choose to investigate the objections offered to amalgam, we shall not go far wrong in this or any other line of study which will add something of value to the common fund of knowledge, if we keep in mind the advice of Polonius to his son,—

> "This above all,—to thine own self be true;
> And it must follow, as the night the day,
> Thou canst not then be false to any man."

